# A qualitative study exploring the lived experiences of patients living with mild, moderate and severe frailty, following hip fracture surgery and hospitalisation

**DOI:** 10.1371/journal.pone.0285980

**Published:** 2023-05-18

**Authors:** Vanisha Patel, Antje Lindenmeyer, Fang Gao, Joyce Yeung

**Affiliations:** 1 Department of Anaesthetics, University Hospital Birmingham NHS Foundation Trust, Birmingham, United Kingdom; 2 Institute of Clinical Sciences, University of Birmingham, Birmingham, United Kingdom; 3 Birmingham Acute Care Research Centre, Institute of Inflammation and Ageing, University of Birmingham Research Laboratories, Birmingham, United Kingdom; 4 Warwick Medical School, Warwick Clinical Trials Unit, University of Warwick, Warwick, United Kingdom; World Health Organization, BELGIUM

## Abstract

It is well recognised that hip fracture surgery is associated with a negative impact on short and long-term post-operative physical health and emotional well-being for patients. Furthermore, these patients are known to be frail with multiple co-morbidities. This study explores how frailty shapes the lived experiences of rehabilitation and recovery for patients who have undergone hip fracture surgery. Semi-structured interviews were conducted with sixteen participants, recently discharged from hospital following hip fracture surgery. Interpretative phenomenological analysis was applied to explore the lived experiences of frail patients and ascertain important themes. Patient experiences were captured in seven overarching themes: 1) the hospital as a place of “safety”, 2) placing trust in others, 3) the slow recovery journey impeded by attitude and support, 4) maintaining autonomy and dignity whilst feeling vulnerable, 5) seeking a new normal, 6) loneliness and social isolation and 7) the ageing body. Based on our study findings, we have been able to suggest a number of opportunities to improve support for frailer patients in finding a new routine to their everyday lives, these include on-going physical and psychological support, information and education and a robust pathway for transition of care into the community. A conceptual thematic diagram is presented which helps to understand the experience and the complex needs of frail older people undergoing hip fracture surgery.

## Introduction

The ageing population presents serious challenges to our health and social care system. There are now 11.4 million people aged 65 or over in the UK and for the general surgical population prevalence estimates range between 10% and 37% for frailty [1, 2]. In the UK, approximately 70 000 to 75 000 hip fractures occur each year with the majority of these occurring in the older person [3]. Clinical outcomes such as mortality and length of stay have long been a measurement of success of patient care for clinicians [4]. Clinical outcomes and patient experience are invariably linked; positive patient experiences have been found to be associated with better clinical outcomes [5, 6]. The NHS Outcomes Framework has reported a role for positive patient experience, the need to measure care as perceived by patients, and the need for the healthcare system to respond and act on such feedback. Thus, they are advocating information gathering about the lived healthcare experiences of patients [7].

Older patients have complex needs, and age alone does not simply define important patient centred care needs. Involving patients themselves in the identification of their care needs allows patient experience to become an important component in the evaluation of quality of healthcare, ensuring patients focused care and overall improving their healthcare journey, by making patients feel more supported and cared for.

Frailty has been defined as a state of increased risk, a distinctive health state related to the ageing process in which multiple body systems gradually lose their in-built reserves resulting in patients having more health deficits [8, 9]. It is a condition characterised by loss of biological reserve, failure of physiological mechanisms and vulnerability to a range of adverse outcomes including increased risk of morbidity, mortality and loss of independence in the perioperative period [10]. Two main theoretical constructs exist in the assessment of frailty, Fried et al’s phenotypic model and the deficit model described by Rockwood and Mitnitski [11, 12]. The former defines five components, exhaustion, weight loss, weak grip strength, slow walking speed and low physical activity. Multi-system dysregulation is considered if three or more of these components are observed. which in the presence of three suggest multi-system dysregulation. The latter quantifies accumulated deficits; it assesses the number of health problems that a patient has accumulated over their lifetime. Frailty assessment brings together information about health deficits and their impact on the patients’ ability to think and do as they please; look after themselves; interact with other people; and move about without falling [8].

Hip fracture patients are well known to be frail with multiple co-morbidities; high pre-operative frailty scores are associated with increased length of hospital stay, 30- and 90-day mortality and likelihood of institutionalisation [13–15]. Moreover, frailty has also been recognised as a predictor of post-operative complications and poor functional outcomes, as well as a risk factor for prolonged hospital stay, institutionalisation and worsening disability, resulting in a vicious cycle [13, 16–18]. In practice, recognising frailty can indicate the need for a holistic approach to treating older patients with complex needs across health and social care [19].

Qualitative studies evaluating the experiences of frail patients have focused on assessing those with chronic medical conditions or undergoing elective surgery [20, 21]. Patients discussed fears included being ignored or feeling imprisoned due to loss of physical capability, cognitive decline leading to dementia and nursing home admission [22]. In addition, patients recognise the physical symptoms of lack of strength, weight loss and risk of falls associated with this chronic disease state but also add that there is a psychological component to being frail in addition to having physical symptoms [23]. Social interaction plays a greater role in maintaining quality of life for frail patients compared to the non-frail [24]. For many frail patients, the greatest priority is to maintain independence, with their well-being centred on their ability to complete everyday tasks [25].

Frail patients undergoing hip fracture surgery are a heterogenous group with respect to co-morbidities, functional status and social support and therefore it is likely that their rehabilitation and recovery experience will vary [26]. Previous studies exploring patient experiences following a hip fracture have outlined the multifaceted experience of recovery. Most studies have focused on the period immediately after injury; including how patients make sense of the acute injury and hospital experience [27–29], communication and information provision [30, 31], interactions with others on the ward and management of acute pain [32, 33]. Other studies have explored more contextual factors such as patients’ experience of the discharge process and transition of care [30, 34], challenges patients experience to rehabilitation [28, 30, 32, 35], as well as the importance of support from family and friends [27, 30, 34, 35]. Long term rehabilitation and recovery are of particular concern to patients as they return home with the aim to minimise loss of independence [36, 37]. To date, qualitative studies have not focused on frailty as a factor that can impact on the lived experiences of patients following hip fracture surgery. Therefore, the aim of our study was to explore the lived experiences of patients living with mild, moderate and severe frailty following hip fracture surgery, with a focus specifically on frail patients’ perceptions of their rehabilitation and recovery experience.

## Methods

### Ethics

The study received approval from the West Midlands Research Ethics Committee. (REC reference number: 16/WM0165) We conducted and reported this study in compliance with the consolidated criteria for reporting qualitative research (COREQ) [38].

### Design

In order to capture patients’ experiences of living with varying degrees of frailty, we conducted a qualitative study based on an interpretative phenomenological approach. Interpretative phenomenology was the chosen methodology as it explores in detail how people make sense of their personal and social worlds [39]. Interpretative phenomenology analysis (IPA) has gained prominence in health and social sciences as a way to understand and interpret topics which are complex and potentially emotional, such as illness experiences [40]. By applying this approach, we aimed to identify, explore and describe the lived experiences of patients following hip fracture surgery, with an emphasis on an individual’s personal perception. IPA focuses on small and homogeneous samples, with participants purposively selected because they have experiences of the phenomena studied. Each participant gives an in-depth reflective narrative of their own experiences from their own perspective. The participants interpret their own experiences and the researcher uses their own interpretation to come to an understanding of the participants experience, thus a two- stage interpretation process was involved known as ‘double hermeneutics’.

### Frailty assessment

We chose to use a deficit model for frailty. The Clinical Frailty Scale (CFS) is a tool combining clinical assessment with objective evaluation of specific domains including comorbidity, function, and cognition to generate a frailty score ranging from 1 (very fit) to 9 (terminally ill). For this study, patients were categorised into four groups 1) Fit (CFS score of 1–3), 2) Mild frailty (CFS score: 4–5), 2) Moderately frail (CFS score: 6) and 3) Severe frailty (CFS score: 7–9) [8, 41]. Frailty status was measured, at interview, 8–12 weeks post hip fracture surgery by the lead researcher (VP).

### Recruitment

Purposive sampling [42] was used to recruit potential participants between December 2016 and July 2017 at Birmingham Heartlands Hospital, University Hospital Birmingham NHS Foundation Trust. The sample aimed to obtain a range of patients of varying age, gender, co-morbidities and baseline frailty status. Participants were eligible to take part in the study if they were aged 65 years and over and underwent hip fracture repair surgery. Patients were excluded if they were unable to consent or unable to partake in the interview due to a speech or language impairment. The researcher (VP), a female clinical doctor, screened the clinical admission database at Heartlands Hospital to identify potential participants. Patients were recruited, face to face, within 72 hours of admissions; the researcher (VP) introduced the study and provided a written information leaflet. Written consent was obtained. Out of thirty eligible participants, seventeen provided informed consent however one patient declined to be interviewed thereafter. Based on IPA methodology, a sample size of between 5 and 10 in-depth interviews is enough to discover the nuances and complexities of people’s lived experiences [43].

### Data collection

The primary researcher (VP) interviewed each participant. An interview guide was planned and created with input from stakeholders including a patient representative (S1 File). This ensured questions were relevant, patient focused and worded in an easy to understand manner. Initial questions were open-ended exploring how they broke their hip, their hospital experience with prompts related to involvement and support for family/carers, relationship with healthcare professionals and dignity/respect. Further questioning explored their experiences and feelings around discharge, sources of support, any changes to relationships with partners, families and health care providers and their rehabilitation and recovery goals. Interviews were conducted by one researcher (VP) to ensure consistency of interview questions throughout the study. At the time of interview, frailty was evaluated using the CFS.

Semi-structured interviews were conducted eight to twelve weeks following discharge at a time and place convenient to patients. In the majority of cases, patients chose to be interviewed following their hospital follow-up appointment. Participants were given a choice of being interviewed alone or accompanied by a relative/friend. Interviews were recorded and transcribed per verbatim for analysis. Field notes were also made during the interviews by VP.

### Data analysis

Analysis was carried out using the steps outlined by Smith et al. for IPA [39]. VP carried out the initial analysis, listening to the transcripts as well as reading and re-reading. Descriptive codes were noted beside the text, leading to identification of the preliminary themes. With the aim of finding connections between these, VP and JY reflected and interpreted these preliminary themes. Similar thematic concepts were grouped together into a cluster leading to development of a hierarchical system with the initial theme being the one most significant to the participant. Once all transcripts had been analysed, variations between accounts of patients who were living with fit, mild, moderately and severely frail (based on the CFS) were investigated using cross-case analysis resulting in the creation of a thematic matrix by two researchers (VP & JY). Development of themes was a circular process, with a focus on suspension of the researcher’s own pre-conceived ideas and judgements surrounding the text by employing individual knowledge and sensitivity surrounding the subject, to obtain a ‘clear’ view of the phenomena, a process otherwise known as ‘bracketing’ [44]. Re-reading of each transcript, review of assimilated themes for each group and further discussion by the research team (VP, JY, AL, FG) generated seven superordinate themes that captured experiences of the participants. In particular, different attitudes and motivators to rehabilitation emerged as an important concern and therefore they were explored further in our analysis, highlighting the similar and contrasting views between patients with differing frailty status. Themes were developed inductively and were not considered to be pre-existing entities lying within the data.

The cumulative research team had extensive knowledge about peri-operative care surrounding hip fracture surgery together with qualitative research experience, ensuring rigour and reflexivity.

Initial analysis involved noting beside the text of each transcript, codes were applied and thereafter categorised into themes using NVivo software version 11 (QSR, Burlington, Massachusetts, USA), this enabled teamworking, audit trail and manageability of data. In the last phase of analysis, a conceptual framework was developed indicating a number of resources and interventions useful to aid with resuming ADLs and improving their recovery journey.

## Results

Sixteen participants between the ages of sixty-five and eighty-eight were interviewed; 11/16 participants were female (Table 1). Participants had a range of co-morbidities including hypertension, chronic obstructive pulmonary disease, atrial fibrillation, diabetes mellitus and osteoarthritis. Eight interviews were carried out with the participant alone, the rest of the participants were interviewed with a family member or partner. Ten interviews were carried out at Birmingham Heartlands Hospital after a clinic follow-up appointment, the rest at the participant’s home. The mean interview time was thirty-two minutes (range 11 to 83 minutes). As outlined above, we analysed variations between patients according to their clinical assessment by the Clinical Frailty Scale. Three patients were evaluated to be fit (1–3), five patients were living with mild frailty (4–5), five patients with moderate frailty (6) and three patients with severe frailty (7+) (Table 1).

**Table 1:** Patient demographics.

	Gender	Age (years)	Clinical Frailty Scale post-surgery^a^	Discharge destination (Living alone)^b^
Patient 1	F	77	6	Home
Patient 2	M	78	3	Partner’s house
Patient 3	M	88	6	Home
Patient 4	F	76	5	Home (alone)
Patient 5	F	68	4	Home (alone)
Patient 6	F	88	7	Intermediate care^c^
Patient 7	F	71	6	Intermediate care^c^
Patient 8	F	65	5	Home
Patient 9	F	80	4	Home
Patient 10	F	88	8	Home
Patient 11	F	72	6	Home (alone)
Patient 12	F	67	6	Home
Patient 13	M	68	5	Friends’ house
Patient 14	F	87	5	Home (alone)
Patient 15	M	84	5	Home (alone)
Patient 16	M	69	7	Home (alone)

^a^ Clinical Frailty Scale–Fit = 1–3, Living with mild frailty = 4–5 living with moderate frailty = 6, living with severe frailty = 7+.

^b^ For patients discharged home and living alone if applicable.

^c^ Intermediate Care is a form of respite care that supports someone to remain in their own home while they recover from an illness, accident or hospital stay

The following themes were identified as being important to frail patients when evaluating their lived experiences in relation to hospital care, rehabilitation and recovery, following hip fracture surgery. A description of each theme is attached with representative patient quotes.

### The hospital as a place of “safety”

The patients living with mild frailty felt the hospital was a ‘safe’ environment which allowed for *“recovery to begin”*. However, they became frustrated with the limitations of the ward environment for example unfamiliarity with the ward layout and the noise and lights contributing to a poor sleep environment. The daily routine of waiting for assistance in washing and visiting the bathroom, receiving pain relief, problems and disturbances from other patients around them limited their recovery. They felt ready to go home and fully recover in their own environment as described by Patient 11 “*Well I’ve never been in hospital in my life*, *and it’s a bit old*, *80 and you’ve never been in your life*. *It was just strange*, *I couldn’t wait to get out*, *that’s why I told them to do it*.*”* However, frailer patients viewed the hospital as a ‘safe’ environment due to close access to bathrooms, support of healthcare professionals during the day and night, physiotherapy support for on-going rehabilitation and presence of other patients providing social support. For them, discharge from hospital was associated with limited support and worry about how they would cope with washing, dressing, cooking and shopping. Patient 12 described her hospital experience: “*I don’t know I think I felt quite safe in the hospital*, *I felt really safe you know well I can’t fall over and I can’t do this*, *I can’t do that and you know I’m okay here but I mean you can’t stay there forever just because you feel safe can you*. *I felt like I’d just been thrown out and I felt like that this time*.*”* Moderately frail patients who lived alone sought practical as well as emotional support to overcome their low confidence and fear of falling again when they got home compared to those that lived with a partner or family members.

Patients with ongoing chronic disease required help in the community; transition of care was viewed to be poor especially by frailer patients who felt that continuity of care was fragmented and transfer of information, medication and support systems were unsatisfactory. There was limited information on the type of surgery and post-operative medical complications and treatment that they had received, medication changes, and no link between the hospital and their GP. Patient and family involvement in discharge planning was ad-hoc and once in the community, identifying sources of help could be challenging. Some patients felt abandoned (*“left to my own devices” (Patient 2)* with no help in organising follow up or review appointments with a medical professional. Discharge planning for Patient 14 involved *“two ladies who deal with this came and they asked these questions and filled in their clipboard thing and*, *erm*, *oh you’ll be all right*, *you’ll be all right*. *You’ll have the half an hour in the morning*, *half an hour at night*. *I said yes and twenty-three hours all on my own*.*”* Patient 15 said that he *“would have liked to have had somebody come along and assess… I don’t know whether I’m progressing well or not…… the physios came just a couple of times to assess me*, *erm*, *to see that I was doing it properly*, *you know*. *But I have no constant physio care at all*.*”* These accounts show how participants’ level of frailty influenced how they perceived the hospital environment and their confidence in returning home.

### Placing trust in others

Healthcare professionals and family/friends were seen as a source of comfort and support for all patients. Patient 5 described the healthcare professionals on the ward, “*They just made you feel at ease really*, *I suppose*”. They were able to provide knowledge and information, this provided reassurance and therefore patients felt they were able to trust them during this time of uncertainty in an unfamiliar environment.

Healthcare professionals were encouraging with rehabilitation, *“when I left hospital I was quite confident*. *I mean*, *we’d had a good ward*. *We’d had good staff and everything but when I went home*, *once the girls (her daughters) had gone back to work*……*I’d lost all that and slowly your confidence goes because you’re not getting the contact*.” Physiotherapists provided motivation and built confidence resulting in a positive attitude to rehabilitation, “*well*, *the physios are very encouraging and they say oh but you’re doing very well*, *you’re doing very well*.*”* (Patient 14) Patient 11 described her attitude to rehabilitation having had some physiotherapy sessions in hospital, “*I think it’s down to me*. *I think it’s down to me really*, *having the confidence to do it*. *And not be frightened*.*”*

The information given by healthcare professionals was often not questioned by frailer patients as they felt that they did not want to cause any trouble and saw them as *“experts”* who “*knew what they were doing*”. However, due to varying physiotherapy information and input, experiences of rehabilitation differed between patients regardless of frailty status.

### The slow journey of recovery

Fitter patients focused on the motivators to recovery which allowed them to engage in rehabilitation. Patient 2 stated in relation to her recovery, “*I do sometimes think to myself*, *“It’s time we went somewhere*, *so we’ll go on the bus*. *I suppose that’s about it really*.*”* Knowledge, support from family/friends and previous experiences shaped their views on their ability to return home. Looking towards the future, they focused on being able to return to their previous physical state. A positive attitude to recovery with a pro-active approach was a prominent theme for success in their rehabilitation goals and recovery. For example, Patient 8 wanted to “*Just get on with it*.*”* The recovery journey was viewed as being slower than anticipated for patients now living with moderate frailty and they were frustrated with this. Prior to their injury they had felt independent, however sustaining a hip fracture and undergoing surgery had resulted in restriction to mobility and activities of daily living. Frustrations were directed at being unable to drive, pick up grandchildren from school, go to the shops and return to work. Patient 1, stated “*I used to do the school run for the little ones*, *but nobody’s shown me how to get in and out of a car*”. Patients felt that limited access to physiotherapy within the community restricted their progress with rehabilitation as expressed by Patient 7 who wanted to start using a stick, *“No because I had physio in Ann Marie’s [rehabilitation centre] and they said that they was going to come here*, *but I’m just walking around with this [zimmer frame]*.*”* As outlined above, physiotherapists were seen to provide practical advice on exercises and improving mobility as well as reassurance. The majority of these patients were self-motivated and understood that they would have to persevere with rehabilitation to improve their own recovery.

### Maintaining autonomy and dignity whilst feeling vulnerable

For patients living with mild frailty, sustaining a hip fracture and having to undergo surgery generated a new state of vulnerability, especially for those that were previously fitter. Patients who were now living with moderate or severe frailty acknowledged some, previous, limited baseline physical health state and independence. For them, restricted mobility after surgery resulted in requiring help with self-care and personal hygiene; a new and challenging experience which made them feel vulnerable. Patients in this situation still wanted to have choices and be able to decide what was right for themselves. Using the bedpan or needing help getting to the bathroom on the ward was embarrassing. Patient 14 described her experience, “*I said oh I want to go to the toilet*. *Argh and they said*, *well we’re very sorry but we can’t do anything [laughter]*. *So you had to hold it*? *No*, *I just had to do it…*. *Oh it was awful*. *I couldn’t believe it”*. These accounts show that patients living with mild and moderate frailty continued to want to maintain dignity and remain autonomous.

Patient 10 had experienced restriction to her activities of daily living prior to sustaining a hip fracture, she explained, *“X had done it all by the time I’d got home*, *so I had no trouble of that*. *But I didn’t need any advice*, *really*, *because I’d done it all before*.*”* In comparison to those living with mild or moderate frailty, those living with severe frailty were familiar with the need to receive help and trust others.

### Seeking a new normal

Most patients living with mild and moderate frailty said that they had been relatively independent and had experienced good health prior to their injury; chronic conditions were well controlled, they had limited physical frailty and were independent in caring for themselves. The impact of the injury had threatened this and therefore many found they wanted to return to their prior state of “normality”. Patient 13 stated, *“Oh yes even though I’m sixty-eight I’m still fairly mobile*, *I haven’t been able to go out as much*.*”* Patient 11 described her fears of falling “*that’s the word*, *frightened*. *I still am*. *I don’t want to go back into hospital*. *Simple as that*. *I’ve tried hard*. *Some days I don’t want to do it [physiotherapy exercises] but I still do it*.*”* This was also echoed by Patient 1, *“I’d lost all that and slowly your confidence goes because you’re not getting the contact*. *I found that devastating because I couldn’t do what I wanted to do*.*”*

Motivation to continue rehabilitation once discharged from hospital was driven by the patients wanting to “return to normality”, which included resuming their daily routine, restoring their previous physical and emotional well-being. Patient 8 lost confidence after her fall, “*It took a knock*, *I didn’t want to go out at first*, *then I thought ‘I’ve got to get out’”*. Patient 1 described previous daily activities which gave them enjoyment “*I like my gardening*. *I do need retail therapy*. *I do need to get out*. *There’s a limit with what I can do in the garden at the moment anyway”*. Patient 11 was dissatisfied with what she was able to physically do *“Now*, *that’s difficult*. *Because yes*, *I’m pleased I’m home*, *but no*, *I’m not satisfied because I can’t do what I want to do”*. The support of a partner/spouse was paramount in being able to return to normal as they provided support, encouragement and motivation. However, the ‘new normal’ which encompassed walking aids, need for care-givers, assistance in washing and preparing meals was recognised and patients were concerned that they might not be able to return to previous physical health, daily routine and independence.

### Loneliness and social isolation

Physical and psychological impairment of sustaining a hip fracture influenced the patients’ ability to socialise and meet family and friends. Patient 5 explained “*I’d like to meet some other older people*, *I suppose*, *like coffee morning and things like that*. *When you’re on your own all day every day*… *I mean*, *I do go out obviously because I get bored*, *but then I can’t walk far so it’s a viscous circle*. For patients now living with moderate and severe frailty, the ageing body, low mood and loss of support from family and friends contributed to their feeling of loneliness. Patient 7 stated “*I’m on my own and things keep passing through my head*.*”* Negative emotions such also impacted on their desire to socialise with others or allow people to visit. For frailer patients, experiences of loneliness and social isolation also changed their attitude to recovery, with some adopting a “why bother?” outlook on rehabilitation. Patient 10 described feeling “*Helpless sometimes*, *yes*, *not being able to do what I want to do*. *Yeah…*.*Oh*, *definitely*. *Yeah*. *I used to be out every day*.*”* For example, Patient 12 was teary during her interview and stated, “*Yes*, *yes sometimes I can just sit and have a good old weep for nothing really*, *well I think it’s nothing but I don’t know*.*”* Patient 7 explained that “*my brother and my sister in law died with cancer*, *so I’ve got nobody (to help)*.*”* Patients described seeking comfort and reassurance from loved ones and healthcare professionals in hospital as well as in the community.

### The ageing body

Sustaining a hip fracture was seen as a sign of ageing and failure of their own body; the limitations in mobility, physical co-morbidities as well as psychological co-morbidities, contributed to views of “an ageing body”. Patients now living with frailty found that their physical frailty, worsened by limited mobility, impacted on their ability to engage with activities of daily living and the social world. Patient 7 described worsening leg ulcers as impacting her recovery and contributing to her limited mobility “*They’re all broken*, *they’re absolutely soaked*. *(in relation to chronic leg ulcers) I told them not to put these socks on because I had them on before and they leaked*, *and I said ‘don’t put them on’*, *because I couldn’t put my slippers on*, *I can’t get used to wearing them*.*”* The impact of existing chronic disease on rehabilitation and recovery was significant for patients now living with moderate and severe frailty. They described how sustaining a hip fracture worsened their disease or led to additional difficulty in managing it. For example, exacerbations of cardiac and respiratory diseases led to prolonged hospital stays and readmission to hospital in some cases. Patients with previous lower limb problems such as knee arthritis or peripheral vascular disease felt that these conditions had become worse, which made engaging with rehabilitation difficult. Worsening of chronic disease for Patient 3 was seen as a result of ageing, a natural process that was inevitable. He described *“after all this upheaval of your body*, *I think it’s silly to take it on…*. *I suppose so*. *I mean*, *I’m 88 so I can’t go on forever…*. *No*, *got to be sensible about it”*. The short and long-term changes to frail participants’ daily activities put a strain on relationships; caregivers had to become involved in helping to manage their health problems when previously they had little input. In some cases, this resulted in the need for long-term adaptations to lifestyle e.g. no longer being able to visit friends and family. Delirium, affected one’s perception of their own health resulting in a negative psychological impact on one’s body. Patient 3 commented: “*Yes*, *because I’d wake up in the night and I didn’t really know where I was*… *well*, *I knew where I was but when I looked around everything was quiet and still*. *I looked up at the railing where the curtain goes and I could see little horses galloping…*. *A bit silly because I didn’t tell anybody about that*.*”* Psychological factors impacted Patient 7, “*I’m on my own and things keep passing through my head*.*”* The psychological factors impeded engagement with rehabilitation and set them back on their recovery journey.

**Fig 1:**
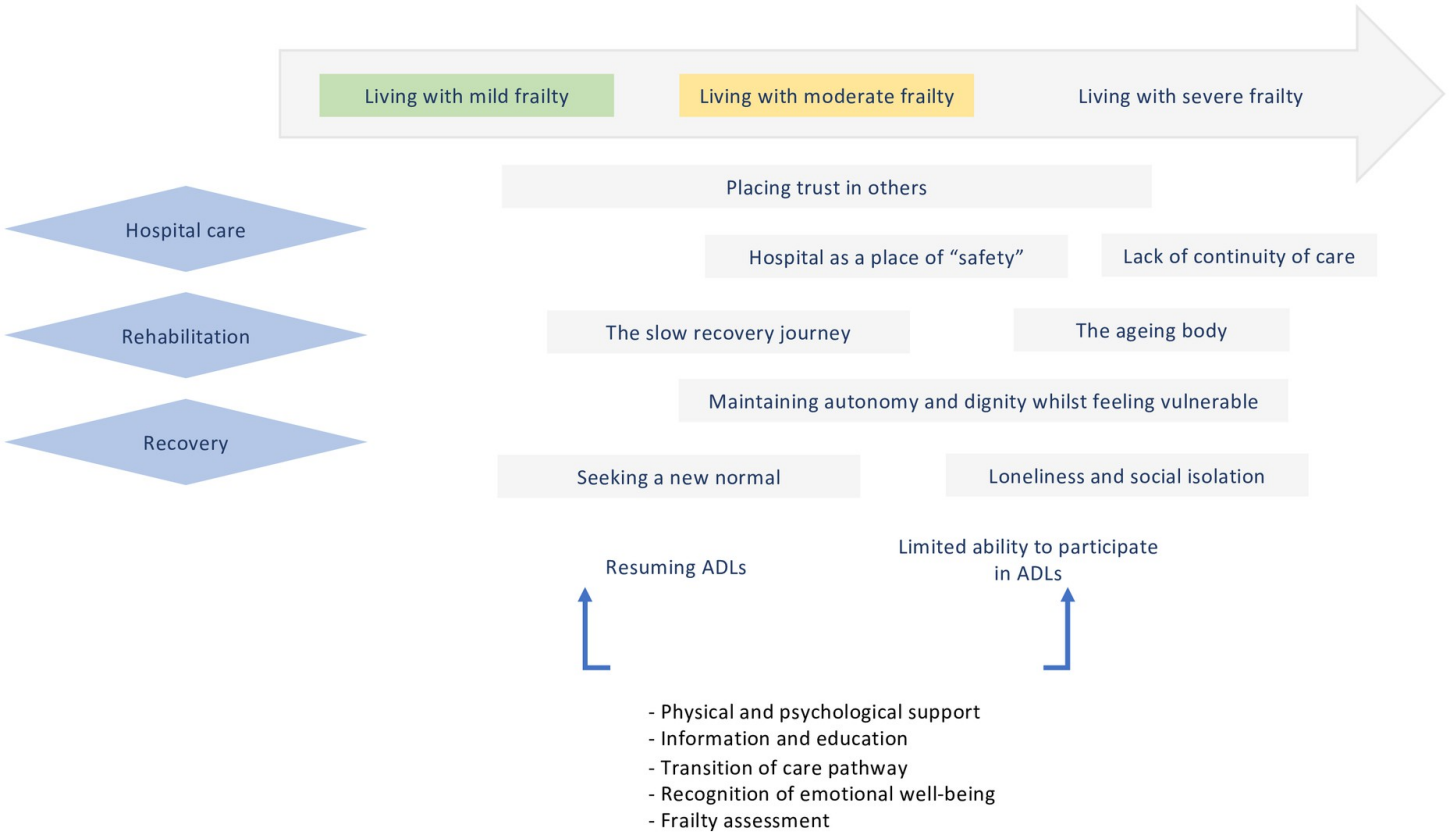
Conceptual thematic diagram of the lived experiences of the frail older person, focusing on hospital care, rehabilitation and recovery, for patients undergoing hip fracture surgery. (ADLs—activities of daily living).

Our conceptual framework (Fig 1) represents an overview of the lived experiences of the frail older person following hip fracture surgery.

## Discussion and implications

This study has highlighted the life-changing impact that hip fracture surgery has on the physical and psychological well-being of frail patients.

The findings reported in this study are consistent with other studies that illustrate patient recovery and rehabilitation post-hip fracture [29, 45–54]. What our study adds is that levels of frailty, influence patients’ lived experiences of recovery from surgery. Furthermore, based on our study findings, we have been able to suggest a number of opportunities to improve support for frailer patients in finding a new routine to their everyday lives (Fig 1).

For frailer patients, placing trust in others was crucial in providing psychological support and comfort which was invaluable in engaging in rehabilitation during a time of uncertainty. Our study found that information and effective communication are central to providing good support and relieving anxiety. It allows patients and their care-givers to participate in shared decision-making and understand the hip fracture ‘pathway’; importantly this makes them feel empowered as well as helping them to understand what to expect in the future and therefore have more realistic expectations and goals for recovery. The current evidence base supports good information provision about their diagnosis, treatment and on-going management, making patients feel comfortable in trusting healthcare professionals and improving overall well-being [27, 30].

Following a hip fracture, the restrictions in activities of daily living experienced by patients lead to the disruption of ‘normal’ life. Similar to findings described by St-Cyr Tribble et al, we have highlighted that, patients ‘seek a new normal’ during their recovery journey [51]. Our participants primarily described factors such as encouragement and support and familiarity of their own surroundings e.g. hospital layout, returning home, in helping to boost motivation and influence their own recovery whilst finding their ‘new normal’. Maintaining normality in everyday life requires psychological, social and physical resources, giving patients the ability to look after themselves, as supported by Claassens et al. [47]. The more resources that are available, the more a positive outlook is adopted by the patient [55]. In frailer patients, acceptability of interdependence should be promoted as well as positive ways of living with frailty. For the frailer patient, fears of interdependence can be reduced through anticipatory care planning and support from communities will promote a sense of belonging, allay social alienation and improve self-worth [56].

In addition, we found that ensuring a smooth transition pathway between the ‘safe’ hospital environment and less controlled environment such as the patient’s home or a rehabilitation unit/nursing home can make patients feel less isolated while ameliorating feelings of vulnerability. Other studies have found that a well-organised transition with adequate support and follow-up can improve quality of life [57]. Some participants felt that discharge plans were inadequate; therefore, early discharge planning, written information about in-patient diagnosis, management and changes to medication, dosette boxes for prescriptions and information regarding community services and follow-up can help in supporting an integrated and co-ordinated care plan to address frailer patients’ individual needs [58].

Furthermore, our study suggests that for frail patients support also includes receiving adequate information and advice for example regarding mobility. There is good evidence that continuity of care can help prevent readmissions, medication errors and improve patient safety resulting in an overall positive patient experience for frailer patients [53].

Sustaining a hip fracture impacted on participants emotional well-being, therefore patients identified as vulnerable may benefit from review and follow-up with a psychologist, this has been shown to help reinforce being aware of ones’ own strengths and limitations and increasing their self-esteem with a reduction in negative feelings [51].

In this study, patients living with moderate and severe frailty, echoed the findings by Taube et al, with barriers to overcoming loneliness including their ageing body, fear of falling and loss of relatives. Additionally, we found that the psychological impact of sustaining a hip fracture together with being frail decreased their desire to socialise with people, in turn having a deleterious effect on mood and further likelihood of social isolation, potentially worsening frailty [59]. As a result, there is a need for social services input for patients and their carers and access to community social networks to reduce loneliness and isolation [60, 61].

This study has provided in-depth personal accounts on the lived experience of hip fracture surgery. Alongside patient safety and clinical effectiveness, patient experience completes the three pillars of quality of care within the NHS [62]. Therefore it is necessary to integrate patient experience into quality improvement and health policy development in order to improve quality of care.

## Strengths and limitations

Our findings provide valuable insight into the needs of frailer patients following hip fracture surgery which is important in guiding healthcare policies. The use of IPA enabled an in-depth thematic exploration of the lived experiences of our participants. Furthermore, semi-structured interviews allowed for in-depth discussion and collection of ‘rich data’. Purposive sampling allowed for frail and non-frail patients to be included and their experiences to be compared.

This is a single-centre study. The hospital that the participants were recruited from serves a diverse multi-cultural population, however only one patient was from an ethnic minority background and the experiences of this group of patients may be different. Whilst we are aware that up to one third of patients experience on-going cognitive dysfunction following hip fracture surgery [63], they were not included in our sample due to the difficulties this would present in conducting in-depth interviews. We acknowledge that the lived experience for these patients and their care-givers would be different, and capturing their views would be a valuable addition to the findings of this study. Interview length was led by the patients, one interview lasted 11 minutes, however the patient felt that this was adequate time for them. Frailty status, was measured post-operatively and therefore some patients may have had on-going ‘deficits’ contributing to their frailty status. However, with limited evidence that frailty is modifiable in this group of patients, measurement of frailty two months post-operatively is likely to reflect a continuing frail state.

## Conclusion

Our findings highlight the impact of different levels of frailty on patients’ experiences during their healthcare journey and therefore it is important to recognise a ‘one-size fits all’ patient pathway will not suffice. Opportunities to improve support for frailer patients highlighted by this study include the need for on-going support for rehabilitation, information and education to shape realistic expectations, a robust pathway for transition of care into the community with access to medical and allied health professionals as well as the recognition and importance of emotional well-being with patients being able to access psychological assessment.

## Supporting information

S1 File Interview question guide. (DOCX)
