# Supplementary material for: A qualitative study exploring the lived experiences of patients living with mild, moderate and severe frailty, following hip fracture surgery and hospitalisation
Source: PLoS One. 2023 May 18;18(5):e0285980. doi: 10.1371/journal.pone.0285980 (PMC10194896; doi:10.1371/journal.pone.0285980)
Supplement: S1 File — (DOCX) [file pone.0285980.s001.docx]

**S1 Fig. Interview question guide**

- Tell me how you broke your hip and what happened after that?
- Can you describe your hospital experience?
- Prompts related to:
  - Pain management
  - Involvement and support for family/carers
  - Relationship with healthcare professionals
  - Dignity/Respect
  - Ward facilities
  - Information
- Were you involved in the discharge planning?
- Did you receive enough support to help you recover and manage your condition at home/rehabilitation? (Medications/ community services/ F/U appointments/ access to support)
- Have you had any difficulty carrying out your usual activities?
- How has this experience affected you emotionally i.e. anxiety/ mood?
- Have you experienced any memory problems?
- Has breaking your hip had an impact on your relationship with relative/friend?
  - Impact/burden on carer +/- coping strategies.
- What information did you/ would you have liked to receive?
- From admission to returning home what is the most important factor you would have changed/improved?
- Is there anything else you would like to add?
